# Nitrergic Pathway Is the Main Contributing Mechanism in the Human Gastric Fundus Relaxation: An In Vitro Study

**DOI:** 10.1371/journal.pone.0162146

**Published:** 2016-09-02

**Authors:** Yang Won Min, Yun Soo Hong, Eun-Ju Ko, Ji-Yeon Lee, Ki Duck Ahn, Je Moon Bae, Poong-Lyul Rhee

**Affiliations:** 1 Department of Medicine, Samsung Medical Center, Sungkyunkwan University School of Medicine, Seoul, Korea; 2 Biomedical Research Institute, Samsung Medical Center, Sungkyunkwan University School of Medicine, Seoul, Korea; 3 Department of Surgery, Samsung Medical Center, Sungkyunkwan University School of Medicine, Seoul, Korea; University of Nevada School of Medicine, UNITED STATES

## Abstract

**Background:**

Human gastric fundus relaxation is mediated by intrinsic inhibitory pathway. We investigated the roles of nitrergic and purinergic pathways, two known inhibitory factors in gastric motility, on spontaneous and nerve-evoked contractions in human gastric fundus muscles.

**Methods:**

Gastric fundus muscle strips (12 circular and 13 longitudinal) were obtained from patients without previous gastrointestinal motility disorder who underwent gastrectomy for stomach cancer. Using these specimens, we examined basal tone, peak, amplitude, and frequency of spontaneous contractions, and peak and nadir values under electrical field stimulation (EFS, 150 V, 0.3 ms, 10 Hz, 20 s). To examine responses to purinergic and nitrergic inhibition without cholinergic innervation, atropine (muscarinic antagonist, 1 μM), MRS2500 (a purinergic P2Y1 receptor antagonist, 1 μM), and N-nitro-L-arginine (L-NNA, a nitric oxide synthase inhibitor, 100 μM) were added sequentially for spontaneous and electrically-stimulated contractions. Tetrodotoxin was used to confirm any neuronal involvement.

**Results:**

In spontaneous contraction, L-NNA increased basal tone and peak in both muscle layers, while amplitude and frequency were unaffected. EFS (up to 10 Hz) uniformly induced initial contraction and subsequent relaxation in a frequency-dependent manner. Atropine abolished initial on-contraction and induced only relaxation during EFS. While MRS2500 showed no additional influence, L-NNA reversed relaxation (*p* = 0.012 in circular muscle, and *p* = 0.006 in longitudinal muscle). Tetrodotoxin abolished any EFS-induced motor response.

**Conclusions:**

The relaxation of human gastric fundus muscle is reduced by nitrergic inhibition. Hence, nitrergic pathway appears to be the main mechanism for the human gastric fundus relaxation.

## Introduction

Several mechanisms have been suggested to explain the association between the symptoms of functional dyspepsia (FD) and underlying pathophysiology [1,2]. Among them, impairment of gastric accommodation accounts for about 40% of cases in FD [3]. Gastric accommodation is activated as the bolus of food reaches the stomach and triggers a reflex largely mediated by intramural intrinsic inhibitory pathways [4].

Over the years, numerous studies with animal models have attempted to explain the neurotransmitters involved in this adaptive relaxation. These studies reached upon an agreement that there is more than one neurotransmitter released by the inhibitory neurons, including nitric oxide (NO) [5–8], vasoactive intestinal polypeptide (VIP) [9–12], and adenosine triphosphate (ATP) [13,14]. Based on animal studies, there is still debate on whether NO predominantly mediates the relaxation [4,15,16], or NO and VIP act as co-transmitters [17–20]. ATP, on the other hand, was addressed as the third agent of inhibitory neurotransmission in the rat gastric fundus by Jenkinson & Reid [21].

On the contrary, only a small number of studies have further substantiated the hypothesis that NO is the major contributor in the non-adrenergic, non-cholinergic (NANC) relaxation of human stomach [22–25]. Although ATP and VIP have also been suggested as responsible agents in the human gastric relaxation, nitrergic pathway seems to the most plausible inhibitory mechanism based on previous *in vivo* gastric barostat experiments [24,25]. However, this hypothesis has not yet been confirmed in physiologic studies. Thus, in this present study, we aimed to clarify the roles of NO in the relaxation of human gastric fundus.

## Methods

### Subjects and tissues

Gastric fundus smooth muscle specimens were obtained from a total of 16 subjects (10 males and 6 females, median age: 55 years, range: 35–83 years) who underwent gastrectomy for gastric cancer at Samsung Medical Center from December 2010 to November 2013. None of these patients had pre-operative radiotherapy, irritable bowel syndrome, or neurological disorders which may alter the basal motility of human stomach. They had no known gastrointestinal disease other than gastric cancer. Diffuse infiltrating stomach cancer, such as Borrmann type IV advanced gastric cancer, was also excluded in this study.

After gastrectomy, the fundus muscle strip samples were taken from the regions free of macroscopic evidence of cancer infiltration. The specimens were incubated in oxygenated modified Krebs-Ringer bicarbonate (KRB) solution (95% O_2_ and 5% CO_2_) at 4°C and immediately transported to the laboratory.

All patients provided written informed consent in accordance with the Declaration of Helsinki. The study protocol was approved by the Institutional Review Board of Samsung Medical Center (No 2009-05-018).

### Isometric force measurements

Experiments were performed *in vitro* with strips of both circular (*n* = 12) and longitudinal (*n* = 13) muscles from human gastric fundus and their mechanical activities were recorded as changes in isometric force. These experiments were conducted using standard organ bath techniques, as previously described in another study by our laboratory [26,27].

Electrical field stimulation (EFS) of intramural nerves was carried out by giving stimuli of various frequencies and durations (0.3 ms in trains of 1–20 Hz for 1–20 s, 150 V) which were applied via the two platinum ring electrodes attached to each strip. The electrodes were connected to a GRASS S88 (GRASS Instruments, Quincy, MA, USA) stimulator.

### Protocols

In the first series of experiments, to understand purinergic and nitrergic roles in the spontaneous contraction-relaxation of gastric fundus muscle under non-cholinergic condition, atropine (a muscarinic antagonist, 1 μM), MRS2500 (a purinergic P2Y1 receptor antagonist, 1 μM), and N-nitro-L-arginine (a nitric oxide synthase inhibitor, L-NNA, 100 μM) were added in a sequential order to the organ bath. Then TTX (1 μM) was administered to inhibit any nerve-mediated contraction. Basal tone, peak, mean amplitude and frequency were observed for 5 minutes in the control state and after serial administration of each drug. The peak amplitude represented the highest measured amplitude, while the basal tone stood for the lowest value measured during the observation time. The amplitude was calculated as the subtracted amplitude between the peak and basal tone. Frequency was defined as the number of contractions per one minute which was measured as the total number of contractions divided by the minutes of observation time. The contractions were expressed in grams for peak, basal tone, and amplitude, while cycles-per-minute (cpm) was used for frequency.

Next, optimal conditions for EFS experiments using human gastric fundus muscles were studied; (i) EFS was applied at various frequencies (1, 5, 10, and 20 Hz) and train durations (1, 5, 10, and 20 s); (ii) recovery of regular contraction was observed after varying durations of EFS (from 10 s to 5 min); and (iii) electrical stimulations with frequencies from 1 Hz to 100 Hz for 20 s were delivered to the specimens immersed in TTX (1 μM)-containing organ bath to define the maximum frequency of EFS causing neurally-mediated contraction.

**Fig 1:**
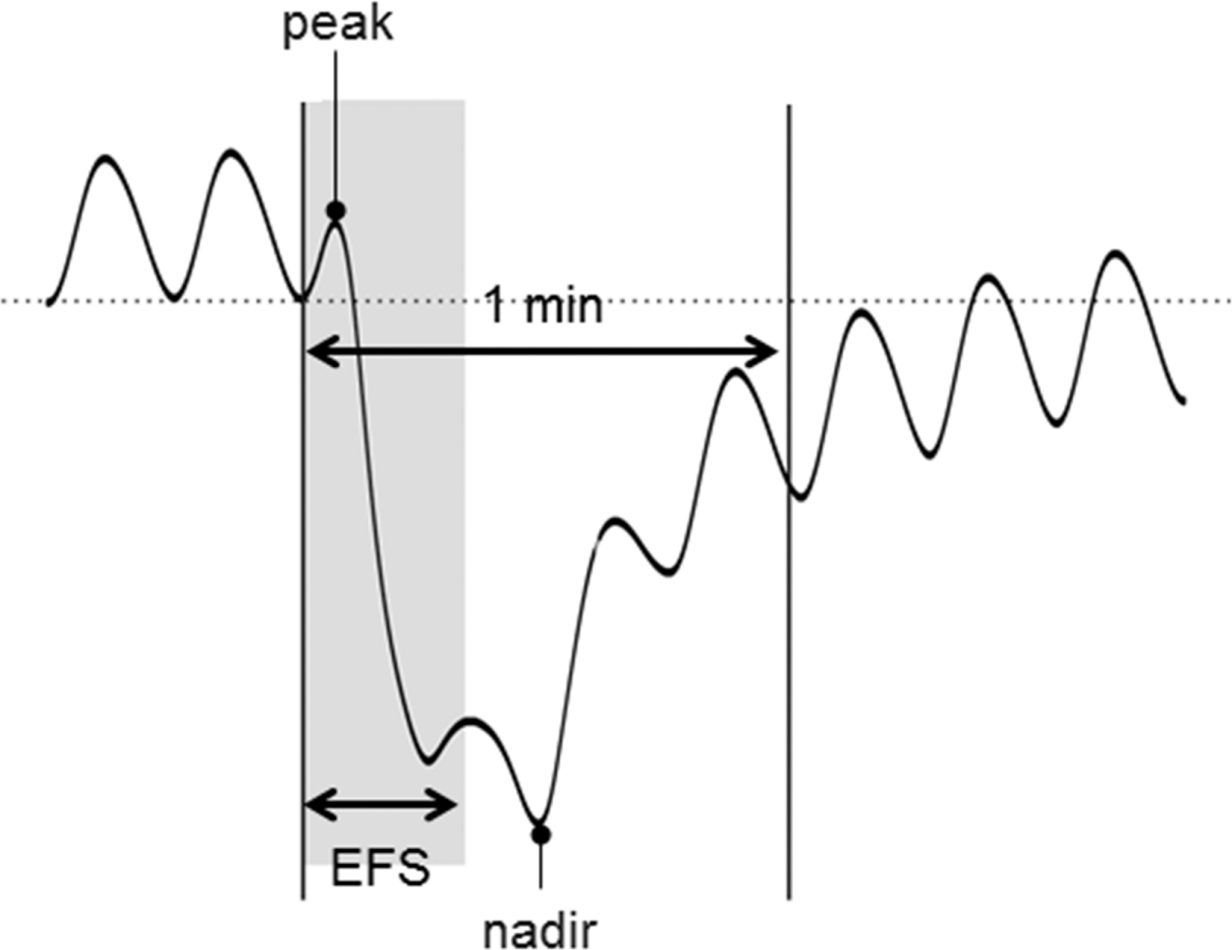
Peak and nadir represent the contractile response induced by electrical field stimulation (EFS). Peak is the highest value, while nadir stands for the lowest value during the first one minute after the initiation of EFS.

Using the optimal study setting obtained from this study (150 V, 0.3 ms, trains of pulses at 10 Hz, and 20 s in duration), the muscle strips immersed in standard organ baths were perfused with atropine (1 μM), MRS2500 (1 μM), L-NNA (100 μM), and TTX (1 μM) sequentially, as in the first set of study for spontaneous contraction. Peak and nadir were measured for 1 minute, including the 20 s of EFS, in the control state and after addition of each of the four drugs. We defined the peak as the highest value and the nadir as the lowest value during the first one minute after the initiation of EFS (Fig 1).

### Immunofluorescence

The fundus from human stomach was isolated and pinned flat on Sylgard coated dish. Tissue was fixed in 4% paraformaldehyde saline solution for 1 hour at 4°C and washed with phosphate buffered saline (PBS). Tissue was dehydrated in successive sucrose solution in PBS in 5%, 10%, and 15% for 1 hour and at 20% overnight. The fundus was cut into pieces approximately 5 mm x 5 mm circumferentially and put into Tissue-Tek Cryomold (Sakura Finetek, Torrence, CA, USA) containing Tissue-Tek OCT solution (1:1 20% sucrose solution and OCT). Tissues in blocks were snap-frozen in liquid nitrogen cooled isopentane and stored at -80°C.

12 μm thin sections were cut on a Leica 3050S cryostat (Leica Microsystems, Wetzlar, Germany) on Vectabond (Vector Laboratories, Burlingame, CA, USA) coated slides. Sections were air dried at room temperature for 1 hour and washed in PBS three times for 5 minutes then blocked with 10% bovine serum albumin (BSA) solution for 1 hour then incubated overnight with anti-nNOS (SC-648, Santa Cruz Biotechnology, Inc, CA, USA) at 1:250 in 0.5% Triton X-100 in PBS overnight in 4°C. Slides were then washed three times for 5 minutes in PBS and incubated in Alexa Fluor 488 donkey anti-Rabbit IgG (1:1000 in PBS, Invitrogen, Carlsbad, CA, USA) for 1 hour at room temperature and washed in PBS (3 times, 5 minutes each) and mounted with glass cover slips with Aqua-Mount (Lerner Laboratories, Pittsburgh, PA, USA). The sections were then visualized with Zeiss LSM150 with the 488 nm laser and acquired in Zeiss LSM 5 Image Examiner and arranged in CorelDraw X4.

### Statistical analysis

Data are expressed as mean ± SEM. The Wilcoxon signed-rank test was used to evaluate the effects of each drug. Multiple comparisons were corrected by Bonferroni’s post-hoc test. *P-*values less than 0.05 were considered significant. Statistical analysis was performed using SPSS version 21 (SPSS IBM, NY, USA).

## Results

### Contributions of purinergic, and nitrergic pathways in the spontaneous contractions of human gastric fundus muscle

**Fig 2:**
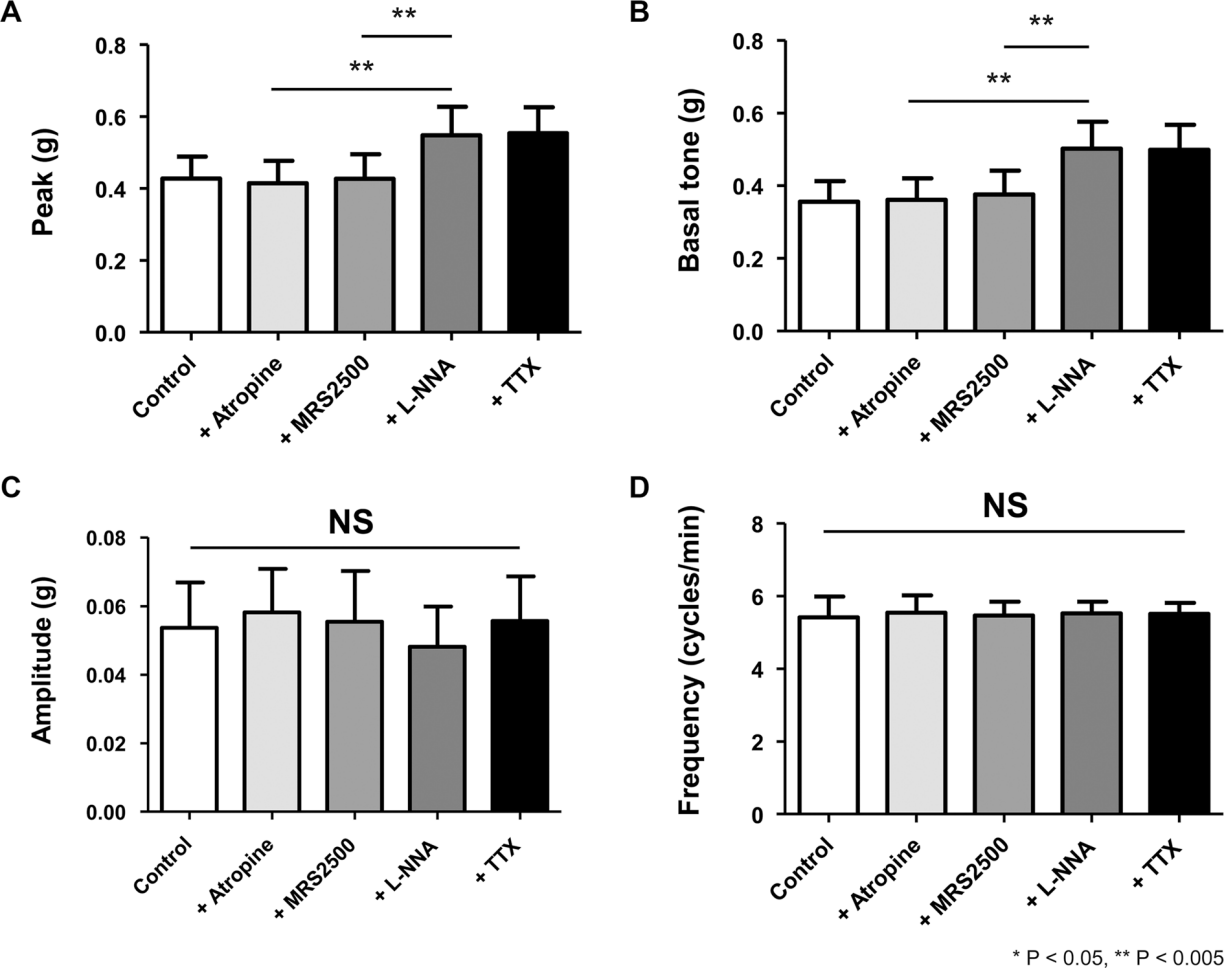
The four parameters of spontaneous contraction in the circular smooth muscle of human gastric fundus. Basal tone and peak amplitude increase to a significant degree by inhibition of NOS. Mean amplitude and frequency, on the other hand, are not affected by inhibition of purinergic and nitrergic pathways.

Stretching muscle strips to a resting tension developed spontaneous phasic contraction, which persisted throughout the experiment. Firstly, we tested the effects of atropine, MRS2500, L-NNA, and TTX on spontaneous contractility. Overall, in the circular muscle, addition of atropine and MRS2500 had no effect on contractility measured by the four parameters mentioned above (Fig 2). However, L-NNA in the mixture increased the peak amplitude (atropine *vs* atropine + MRS2500 + L-NNA, 0.42 ± 0.06 *vs* 0.55 ± 0.08, *p* = 0.024). A similar tendency was observed for basal tone as L-NNA reversed the intrinsic relaxation component and increased basal tone to a significant degree (atropine *vs* atropine + MRS2500 + L-NNA, 0.36 ± 0.06 *vs* 0.50 ± 0.07, *p* = 0.018). In terms of the amplitude and frequency, there was no significant change regardless of the administration of L-NNA. TTX did not further alter spontaneous contractility in the presence of these drugs.

**Fig 3:**
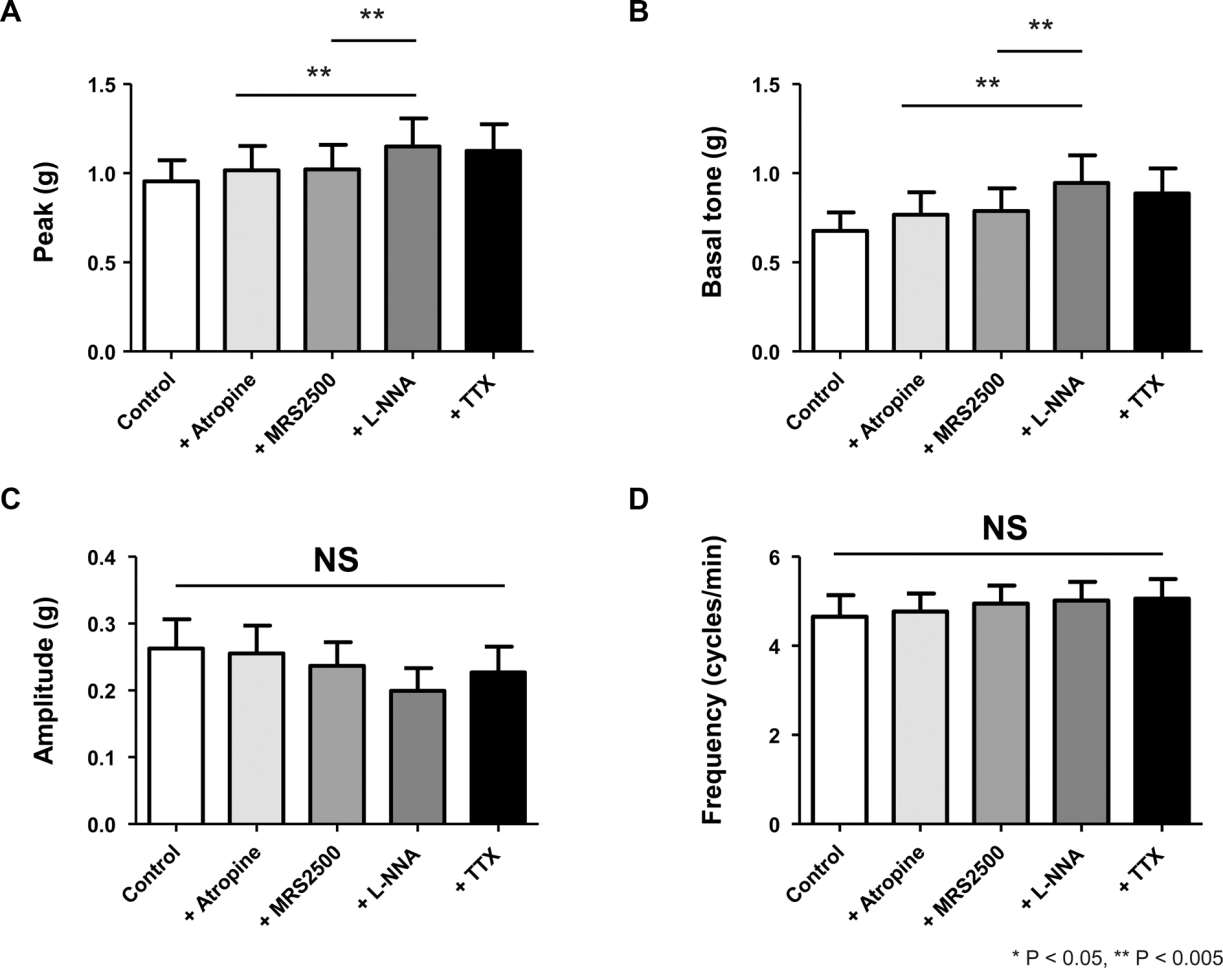
The four parameters of spontaneous contraction in the longitudinal smooth muscle of human gastric fundus. As in the circular muscle, basal tone and peak amplitude increase significantly by L-NNA, but mean amplitude and frequency are unchanged by serial administration of MRS2500, and L-NNA.

The results were consistent in the gastric longitudinal muscles. Under the same non-cholinergic condition, MRS2500 did not significantly influence spontaneous contraction. L-NNA, on the other hand, increased the peak (atropine *vs* atropine + MRS2500 + L-NNA, 1.02 ± 0.14 *vs* 1.15 ± 0.16, *p* = 0.012) and basal tone (atropine *vs* atropine + MRS2500 + L-NNA, 0.77 ± 0.13 *vs* 0.95 ± 0.15, *p* = 0.012) (Fig 3). Amplitude and frequency were not affected. TTX had no additional effect on spontaneous contraction.

### Effects of EFS frequency and duration on human gastric fundus muscle

In our experiments, human gastric fundus muscle in response to EFS commonly elicited early phasic contraction and delayed relaxation, which depended on the frequency and duration of EFS. In this set of experiments of EFS with various frequencies (1 Hz, 5 Hz, 10 Hz, and 20 Hz), EFS-induced relaxation was found to be frequency-dependent up to 10 Hz. The nadir of relaxation between 10 and 20 Hz was not different. In addition, since the muscle strips were not tolerable to repeated exposure to EFS of frequencies higher than 20 Hz, it was impossible to continue further tests. When strips of gastric fundus muscle were also exposed to EFS with varying durations (1 s, 5 s, 10 s, and 20 s), the nadir dropped with a duration-dependent manner.

### Frequency of EFS and nerve-mediated relaxations in human gastric fundus muscle

The EFS-induced relaxations were abolished by TTX (1 μM) when the frequency was 20 Hz and below. Frequencies of 50 Hz and higher (70 Hz, and 100 Hz) produced contractions, rather than relaxations, which were not TTX-sensitive.

### Contributions of purinergic, and nitrergic pathways in the EFS-induced relaxations of human gastric fundus muscle

**Fig 4:**
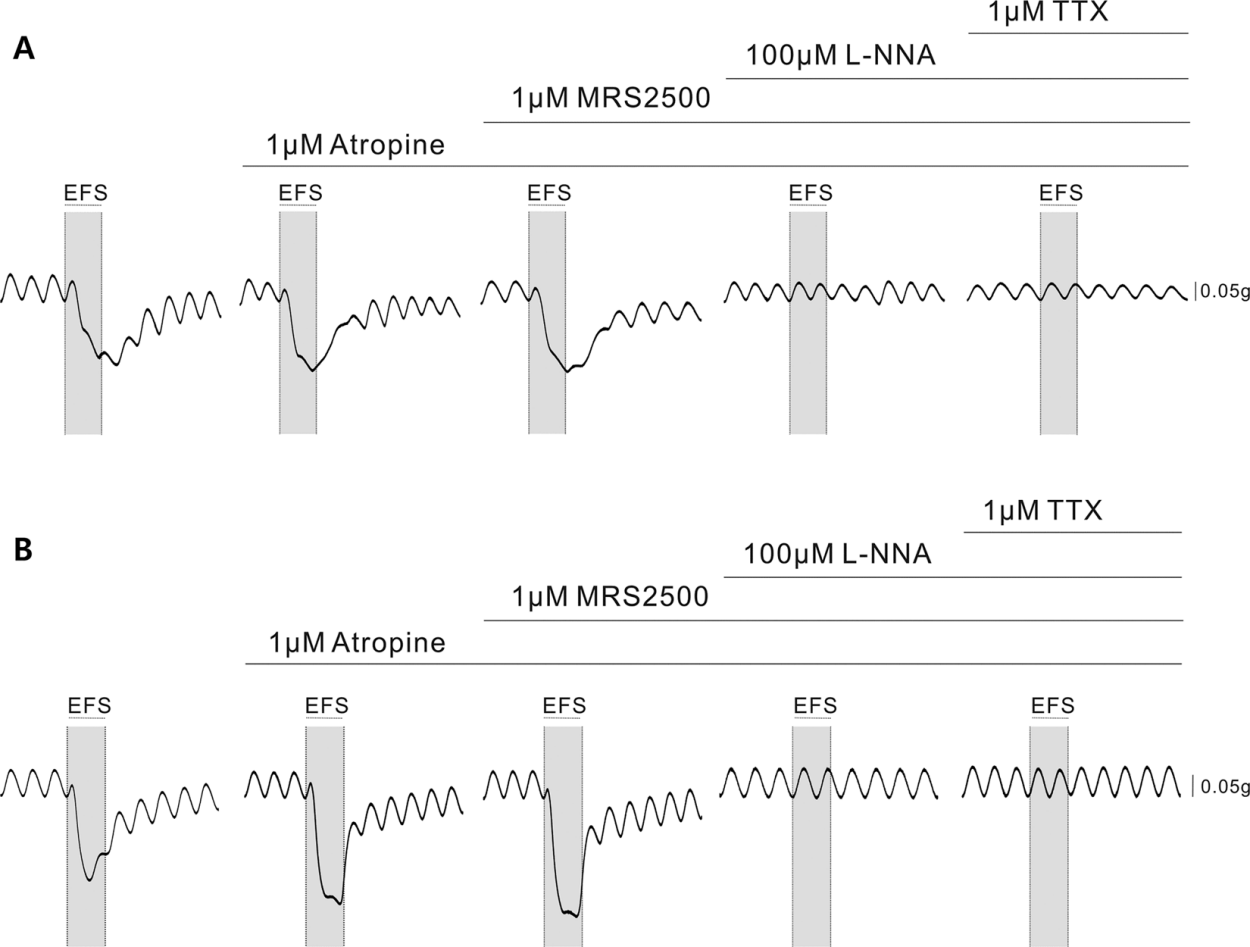
Serial administration of atropine, MRS2500, L-NNA, and TTX under EFS on circular and longitudinal human gastric fundus muscles (10 Hz, 150 V, 0.3 ms and 20 s). (A) A representative tracing of EFS-evoked motor responses of circular muscle. NOS inhibitor reverses the EFS-induced relaxation. (B)A typical tracing of EFS-evoked motor responses of longitudinal muscle. EFS-induced relaxation is also reversed by L-NNA.

Based on above experiments, we concluded that electrical parameters to evoke optimal nerve-mediated relaxation in human gastric fundus are 10 Hz for 20 s. With this preset condition for EFS, we tested the effects of pharmacological treatment using atropine, MRS2500, L-NNA and TTX. Fig 4 shows the typical tracings of responses to the four drugs under EFS. Overall, in both layers of the gastric fundus smooth muscle, electrical field stimulation induced initial contraction and subsequent relaxation. The degree of EFS-induced relaxation was increased by atropine. The tracing pattern after the addition of MRS2500 did not exhibit any significant change from that produced by atropine. However, the relaxation was antagonized by L-NNA, which abolished relaxation produced by previous drugs. TTX blocked any nerve-mediated contractile response.

**Fig 5:**
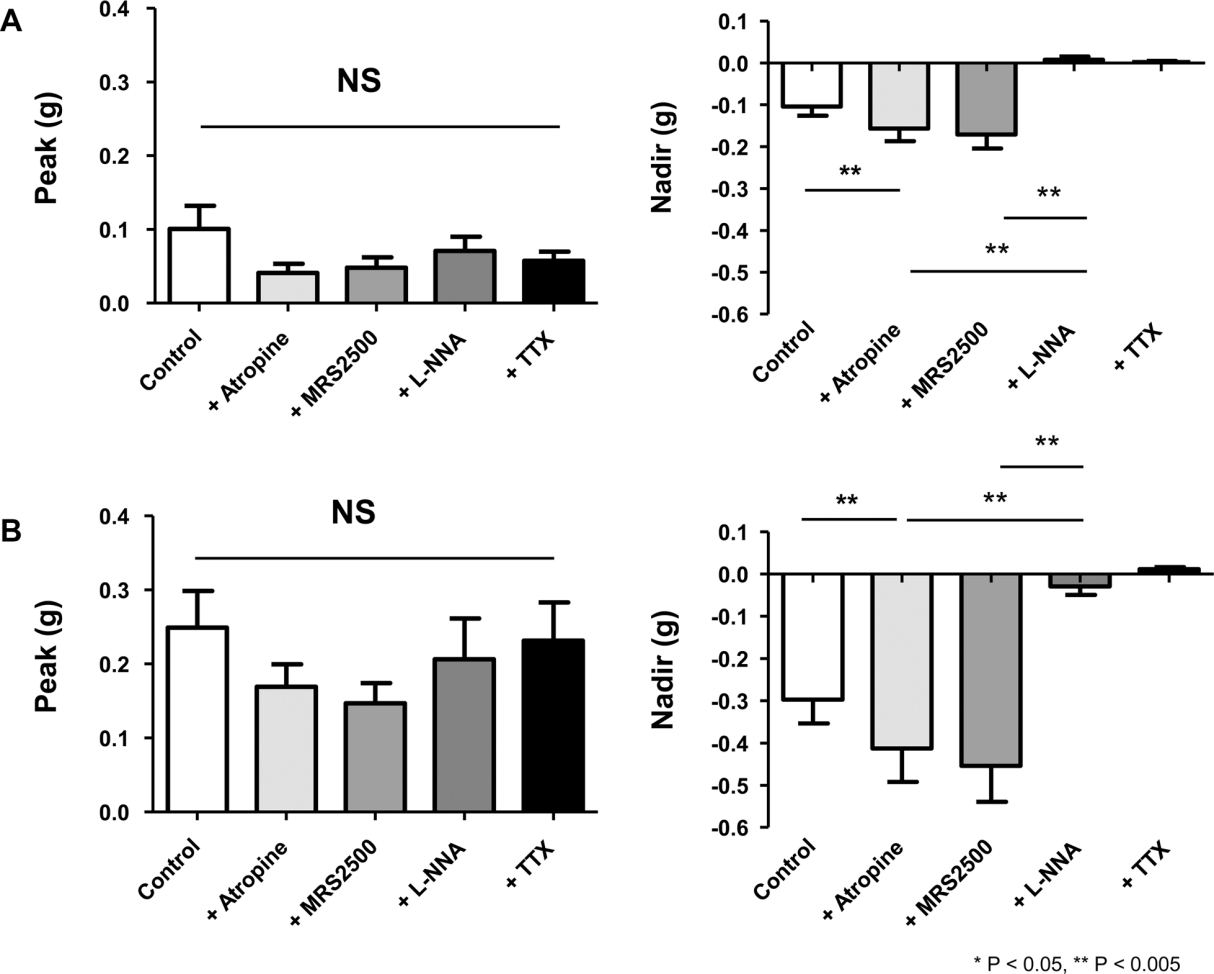
Pharmacological responses, measured by peak and nadir values, to purinergic and nitrergic inhibitors under non-cholinergic condition. (A) In circular muscle, the peak contraction is not changed by addition of MRS2500 and L-NNA to atropine-containing media. The relaxation effect of atropine on nadir is not affected by MRS2500, but reversed significantly by addition of L-NNA. (B) As in the circular muscle, the peak contraction of longitudinal gastric smooth muscle is not affected by MRS2500 and L-NNA. The nadir is reversed significantly by addition of L-NNA.

The peak and nadir in circular and longitudinal muscle strips during EFS are represented in Fig 5. In the circular muscle (*n* = 12), none of the following drugs, atropine, MRS2500, and L-NNA significantly reduced the peak amplitude *p* = 0.061, *p* = 0.179, and *p* = 0.159, respectively) (Fig 5A). On the contrary, when atropine was added to the control, its anticholinergic effect accentuated the relaxation and resulted in a more negative value of nadir, from -0.10 ± 0.02 g to -0.16 ± 0.03 g (*p* = 0.012). MRS2500 did not reverse the relaxation caused by atropine. However, as L-NNA was superimposed on MRS2500-containing media, the nadir was reversed from -0.16 ± 0.03 g to 0.01±0.01 g by inhibiting NO-mediated relaxation (*p* = 0.012). TTX abolished any existing electrically-induced neurogenic response and the nadir of TTX-treated specimens was not statistically different from that of L-NNA-treated muscle strips. In longitudinal muscles (*n* = 13), as in the circular muscles, addition of atropine, MRS2500 and L-NNA failed to reduce the peak contraction during EFS (Fig 5B). The nadir, on the other hand, more deepened from -0.30 ± 0.06 g to -0.41 ± 0.08 g (*p* = 0.03), as atropine blocked contraction which was not affected by MRS2500, but significantly attenuated by L-NNA from -0.41 ± 0.08 g to -0.03 ± 0.02 g (*p* = 0.006).

### Neuronal NOS (nNOS) immunohistochemistry in human fundus smooth muscle

**Fig 6:**
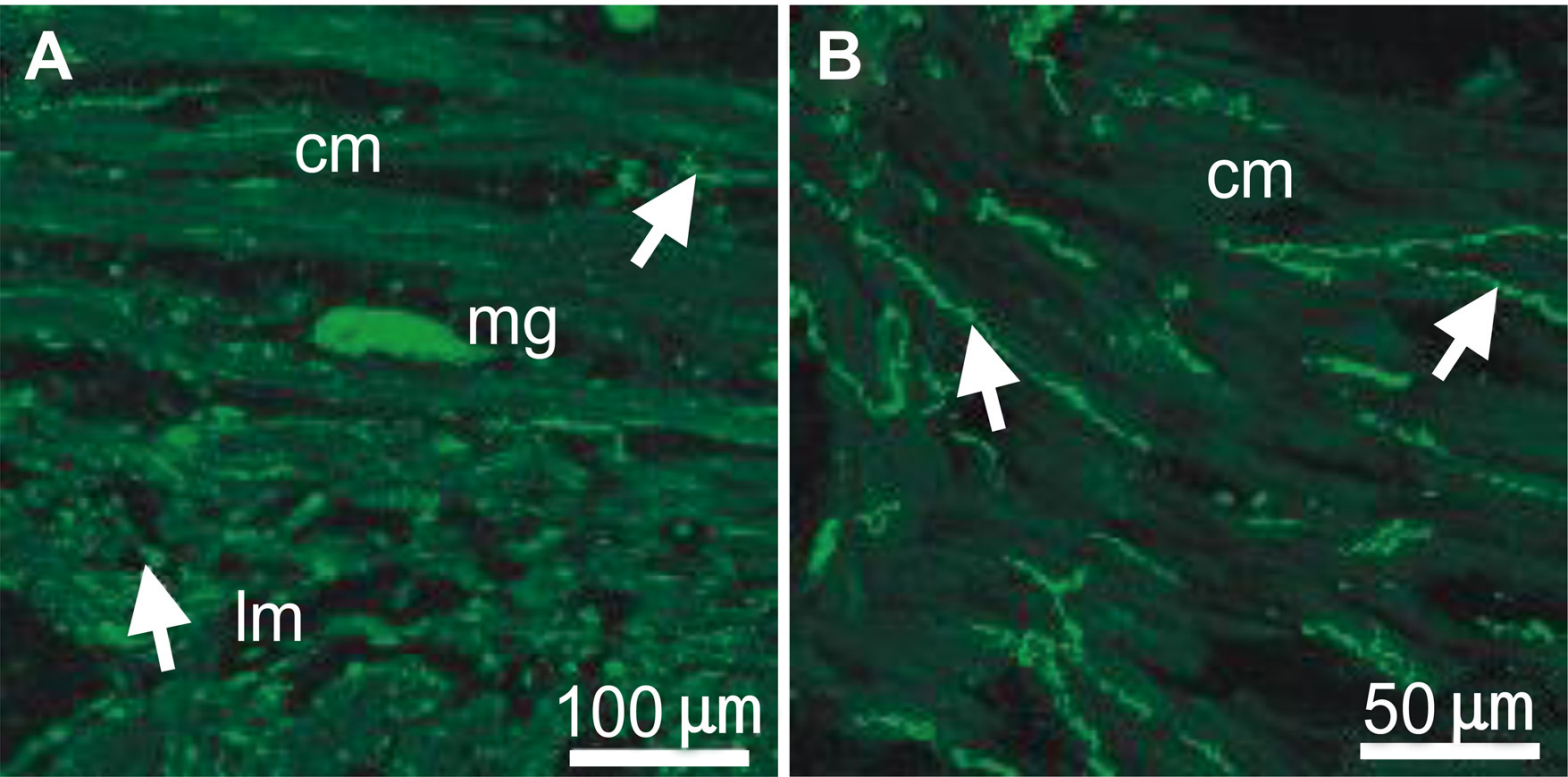
Immunohistochemical localization of neuronal nitric oxide synthase (nNOS) in human gastric fundus. (A) A myenteric ganglion (mg) and nNOS+ nerve fibers (arrows) in a cryostat cross section through the circular (cm) and longitudinal muscle (lm) layers. (B) nNOS+ nerve fibers at a higher magnification running in parallel with the circular muscle (cm) fibers and are varicose in nature (arrows). Scale bars are as indicated in each panel.

NO in the gastrointestinal tract is believed to be synthesized by a nNOS [28,29]. The presence of nNOS in the human fundus was examined by immunohistochemistry. The nNOS+ myenteric ganglion and nerve fibers were present throughout the circular and longitudinal muscle, as well as the myenteric plexus (Fig 6). Long and slender immunostaining was observed intramuscularly throughout the thickness of the muscle running parallel with the circular muscle. The longitudinal muscle layer also displayed immunopositivity as well as myenteric ganglia at the level of the myenteric plexus.

## Discussion

Even now, six decades after the first introduction of the concept NANC relaxation in human gastric fundus muscle [30], there is only limited explanation for the mechanism of how the proximal part of human stomach relaxes and serves as a reservoir during food ingestion [4,5,9,10,13–21]. Animal studies have demonstrated that gastric accommodation is a complex phenomenon induced by multiple neurotransmitters [31]. So far, NO [5–8,22], VIP [9–12], and ATP [13,14,21] have been considered to be the most plausible candidates. Among these inhibitory neurotransmitters, previous studies on animals have provided evidence that mainly NO, and possibly ATP, take part in the early phase of relaxation. VIP, on the other hand, is suspected to be more responsible for sustained relaxation [32]. However, since little is known about how human gastric fundus relaxes as opposed to other animals, our study aimed to understand the physiology of human gastric accommodation. As previous studies have suggested NO to be the major contributing inhibitory neurotransmitter in the stomach, as well as throughout the gastrointestinal tract, a greater portion of those pertaining to the subject has attempted to explain the role of nitrergic pathway in the relaxation of gastric fundus [22–25]. Tonini et al. [22] elucidated that NO and VIP have different roles in the human gastric fundus relaxations: NO being accountable for relaxation evoked by low frequency electrical stimulation and VIP for high frequency stimulation-induced relaxation. In addition, a few *in vivo* experiments on healthy adults have shown that NOS inhibitor, N^G^-monomethyl-L-arginine (L-NMMA), blunts postprandial gastric accommodation by demonstrating a decrease in change of gastric volume after meal ingestion using either 99mTc-single-photon-emission computed tomography imaging [23] or gastric barostat [24,25]. In the same context, we investigated the inhibitory influences of NO in the early relaxation of the proximal human stomach in this study. As the former *in vitro* study included a small number of subjects and only the circular muscle, we supplemented our experiments with more specimens from both layers of human gastric fundus.

Initially, spontaneous tonic contractions of both circular and longitudinal human gastric smooth muscle were observed. Under non-cholinergic environment, the basal tone and peak amplitude were unchanged by MRS2500, but increased to a significant level in the presence of L-NNA in both muscle layers. It indicates that the relaxation component of spontaneous contraction is mediated primarily by nitrergic pathway, rather than purinergic pathway. However, as L-NNA had little effect on the mean amplitude, the change in peak amplitude can be presumed to be the result of increased basal tone.

Also in this study, optimal experiment conditions for EFS of human gastric fundus muscle were determined by reviews of contraction and relaxation patterns evoked by various frequencies and durations of electrical stimulation. Our results suggest that motor responses of human gastric fundus muscle are mediated by neural pathway at lower frequencies (≤ 20 Hz), while muscle strips are directly stimulated by EFS of higher frequency (≥ 50 Hz). In addition, the degree of EFS-induced relaxation was found to have a positive correlation with the duration of stimulation up to 20 s. EFS for longer duration than 20 s did not accentuate relaxation. Thus, we concluded that the optimal condition maximally acceptable for EFS study was 10 Hz for 20 s. The outcomes of these preliminary experiments can be expected to be used for subsequent EFS studies using human stomach muscles.

The specimens of gastric fundus were also exposed to the purinergic receptor antagonist and NOS inhibitor under EFS. Electrical stimulation with isolated trains in non-cholinergic conditions induced TTX-sensitive, short-lasting, frequency-dependent relaxations. Perfusion with L-NNA yielded an increase in nadir, and even reversed it by causing contraction in the circular layer, of the EFS-induced relaxation in the gastric smooth muscle by suppressing the inhibitory pathway. The peak and nadir values of L-NNA-treated muscle strips were not significantly different from those of TTX-treated specimens, indicating that inhibition of NOS alone almost completely blocked any nerve-mediated relaxation. Therefore, NO seems to be the major neurotransmitter in the short-lasting relaxation induced by train stimulation and additionally in the non-cholinergic relaxation of human gastric fundus muscle. However, to fully reverse the relaxation, the possibility of ancillary mechanisms, such as VIPergic pathway, should also be sought in further studies.

The present study has a few strengths. Firstly, preliminary studies defined a setting which enables the muscle specimens to display their optimal and physiologic contractile patterns. Following experiments were conducted with such condition, thereby providing more reliability. Secondly, to the best of our knowledge, it is the first EFS study to distinctly demonstrate that NO has a pivotal role in human gastric fundus relaxation in both circular and longitudinal smooth muscle layers. Moreover, our results show that non-nitrergic pathway was not significantly involved in the relaxation of fundus in human species. In conclusion, we demonstrated that NO predominantly mediates the relaxation of proximal human stomach and that non-nitrergic pathway, including purinergic neurotransmission, has only limited role in the NANC relaxation.

## Supporting Information

S1 Dataset Isometric force measurement dataset. (XLSX)
